# In Real Life, Low-Level HER2 Expression May Be Associated With Better Outcome in HER2-Negative Breast Cancer: A Study of the National Cancer Center, China

**DOI:** 10.3389/fonc.2021.774577

**Published:** 2022-01-17

**Authors:** Yiqun Li, Nilupai Abudureheiyimu, Hongnan Mo, Xiuwen Guan, Shaoyan Lin, Zijing Wang, Yimeng Chen, Shanshan Chen, Qiao Li, Ruigang Cai, Jiayu Wang, Yang Luo, Ying Fan, Peng Yuan, Pin Zhang, Qing Li, Fei Ma, Binghe Xu

**Affiliations:** Department of Medical Oncology, National Cancer Center/National Clinical Research Center for Cancer/Cancer Hospital, Chinese Academy of Medical Sciences and Peking Union Medical College, Beijing, China

**Keywords:** HER2-low, clinicopathological features, metastatic breast cancer, survival, prognosis

## Abstract

**Background:**

To characterize the clinical and pathological features and survival of patients with human epidermal growth factor receptor 2 (HER2)-low breast cancer in China.

**Methods:**

The China National Cancer Center database was used to identify 1,433 metastatic breast cancer patients with HER2-negative disease diagnosed between 2005 and 2015. Clinicopathological features, survival, and prognosis information were extracted. Overall survival (OS) was estimated using the Kaplan–Meier method and compared using the log-rank test. Prognostic factors associated with OS were analyzed using Cox regression model with 95% confidence interval (95% CI).

**Results:**

There were 618 (43.1%) and 815 (56.9%) HER2-low and HER2-zero tumors out of 1,433 tumors, respectively. The proportion of hormone receptor (HR)-positive tumors was significantly higher in HER2-low tumors than in those with HER2-zero tumors (77.8% vs. 69.2%, *p* < 0.001). Patients with HER2-low tumors survived significantly longer than those with HER2-zero tumors in the overall population (48.5 months vs. 43.0 months, *p* = 0.004) and HR-positive subgroup (54.9 months vs. 48.1 months, *p* = 0.011), but not in the HR-negative subgroup (29.5 months vs. 29.9 months, *p* = 0.718). Multivariate regression analysis revealed that HER2-low tumors were independently associated with increased OS in HER2-negative population (HR: 0.85, 95% CI: 0.73–0.98, *p* = 0.026).

**Conclusion:**

Our findings demonstrate that HER2-low tumors could be identified as a more distinct clinical entity from HER2-zero tumors, especially for the HR-positive subgroup. A more complex molecular landscape of HER2-low breast cancer might exist, and more precise diagnostic algorithms for HER2 testing could be investigated, thus offering new therapeutic targets for breast cancer treatment.

## Introduction

Human epidermal growth factor receptor 2 (HER2) is a prototype oncogene that belongs to the HER (EGFR,ErbB) family (1). HER2-positive breast cancer, which accounts for about 15%–20% of all breast cancers, is associated with a more aggressive clinical course and a poor prognosis compared with hormone receptor (HR)-positive, HER2-negative breast cancer (2, 3). Over the past 2 decades, the management of HER2-positive breast cancer has changed dramatically due to the development of anti-HER2 agents, which significantly improved the outcomes of HER2-positive breast cancer patients (4, 5).

Currently, HER2 positivity in breast tumors is defined by the overexpression of HER2 protein measured using immuno-histochemistry (IHC3+) and/or *in situ* hybridization (ISH) (HER2 gene copy number ≥6 or a HER2/CEP17 ratio ≥2.0) (6, 7). Among the 80%–90% HER2-negative breast cancers, a low to moderate expression of HER2 (IHC1+ or IHC2+/ISH-negative) still exists and such tumors are identified as HER2-low tumors (8). Traditionally, tumors classified as HER2-negative are not targetable with conventional anti-HER2 therapies (9). In recent years, however, two HER2-targeted antibody–drug conjugates (ADCs), trastuzumab deruxtecan (T-DXd) (10) and trastuzumab duocarmazine (SYD985) (11), have shown promising antitumor activity in patients with HER2-low breast cancer, thus offering novel therapeutic options for HER2-low tumors and shifting the attention of physicians toward this particular subset of patients (12, 13).

To date, robust studies focusing on HER2-low breast cancer in China are lacking. Few studies from western countries combining HER2-low breast cancer patients from different datasets or clinical trials have yielded varying results (14–16). In this study, we aimed to characterize the clinicopathological features, survival, and prognosis of HER2-low tumors in metastatic breast cancer (MBC) based on the China National Cancer Center database and compare with HER2-zero tumors. Analyses were also performed by HR status and HER2 IHC status.

## Methods

### Patients

Medical records of breast cancer patients treated at the China National Cancer Center were retrospectively reviewed. The China National Cancer Center database was used to identify MBC patients diagnosed between January 2005 and December 2015. Patients were included if they met the following criteria: (i) Histologically confirmed breast cancer with reliable estrogen receptor (ER), progesterone receptor (PgR), and HER2 status, reviewed and reported by two independent breast cancer pathologists from the pathology department of the China National Cancer Center. According to the most updated guidelines established by the College of American Pathologists (CAP), ER/PgR positivity were defined as ≥10% positive tumor cells with nuclear staining by IHC and then ≥1% after April 2010. (ii) HER2-negative breast cancer. HER2 status was assessed by IHC and/or ISH based on the primary tumor sample. HER2 negativity was defined as IHC scoring 0~1+ or IHC2+, but without ISH amplified based on the most recent version of American Society of Clinical Oncology (ASCO)/CAP guidelines at MBC diagnosis. The terms HER2-zero (IHC0) and HER2-low (IHC1+/IHC2+ with negative ISH) were adapted in this study. (iii) Recurrent or metastatic breast cancer. Patients with unknown or equivocal HER2 status were excluded. Demographics of patients, clinicopathological features, sites of disease recurrence, and survival information were extracted. Overall survival (OS) was defined as the time from initial metastatic diagnosis to the date of death from any cause or last follow-up.

### Statistical Analysis

Clinical and pathological features of patients were summarized and stratified by HR status and HER2 IHC status and were compared across groups using chi-square or Fisher’s exact test, where appropriate. Survival curves were constructed using the Kaplan–Meier method and compared between groups using the log-rank test. Subgroup analyses were performed for comparing the differences in age, performance status, disease stage at primary diagnosis, HR status, histology, and metastatic sites between HER2-low and HER2-zero groups. Prognostic factors for OS were analyzed by Cox regression model with 95% confidence interval (95% CI). All statistical analyses were performed using the SPSS 23.0 software (SPSS Inc., Chicago, IL, USA).

## Results

### Baseline Patient Characteristics

**Figure 1:**
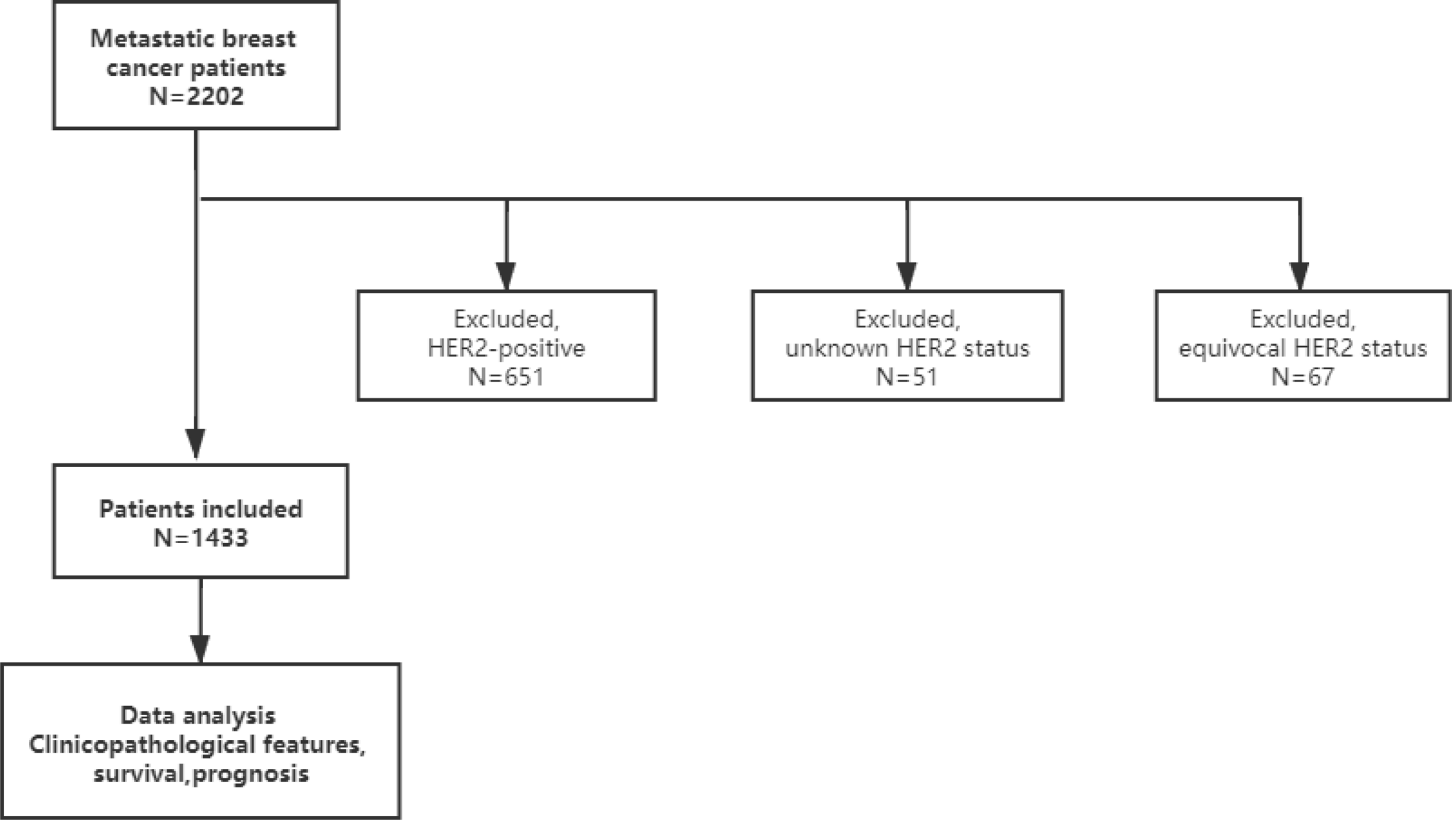
Study flowchart.

We identified 2,202 patients with MBC diagnosed between January 2005 and December 2015 at the National Cancer Center, China. Patients with HER2-overexpressed (n = 651), unknown HER2 status (n = 51), and equivocal HER2 status (n = 67) were excluded (**Figure 1**).

**Figure 2:**
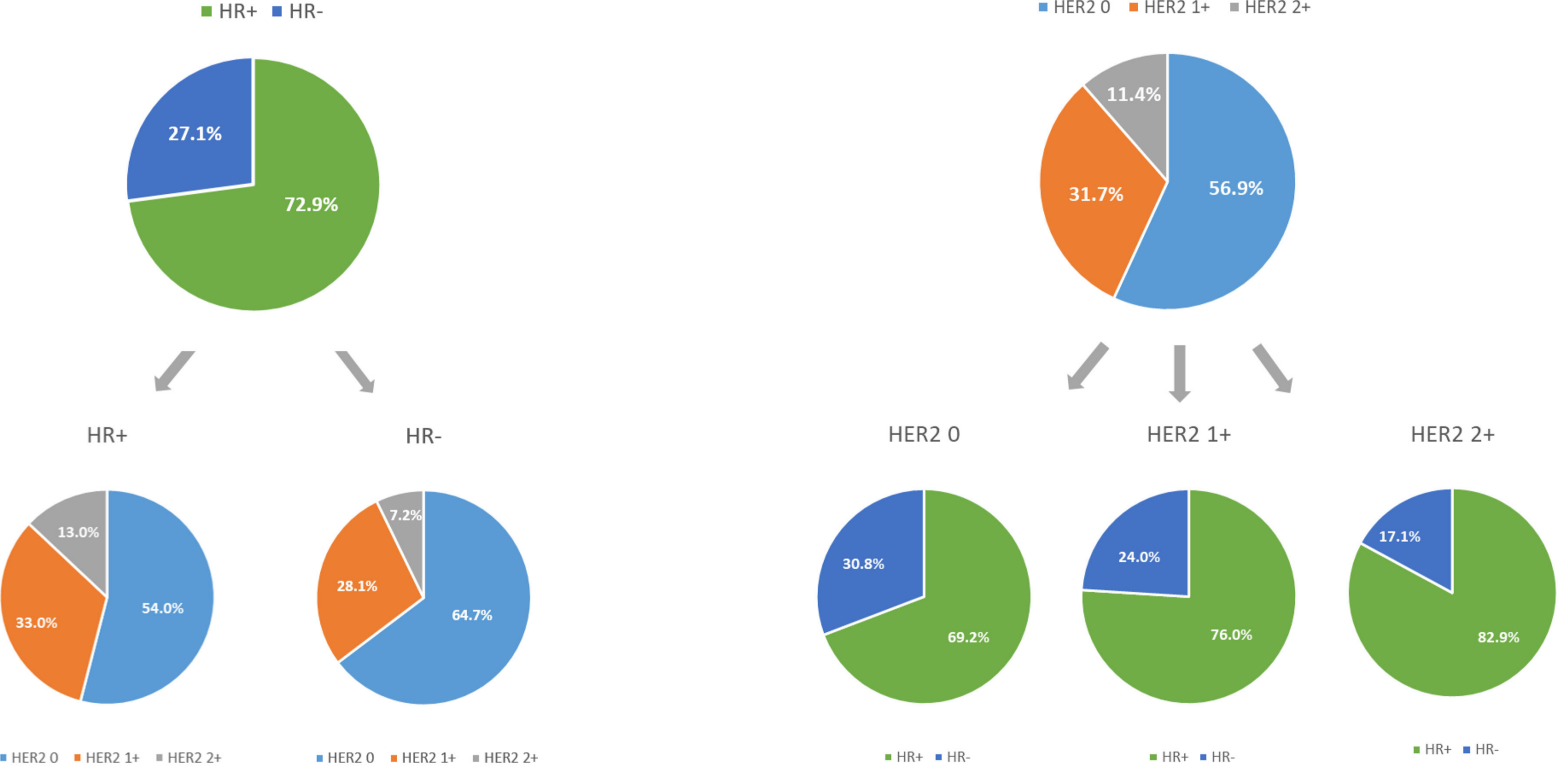
The compositions of human epidermal growth factor receptor 2 (HER2)-negative population by hormone receptor (HR) status and HER2 immunohistochemistry (IHC) status.

Of the 1,433 HER2-negative patients included in this study, 618 (43.1%) had HER2-low tumors and 815 (56.9%) had HER2-zero tumors (**Table 1**). Of the 618 patients with HER2-low tumors, 454 (31.7%) were HER2 IHC1+ and 164 (11.7%) were HER2 IHC2+ (**Figure 2**). When stratified by HR status, 1,045 (72.9%) patients had HR-positive disease and 388 (27.1%) had triple-negative disease (**Table 1**).

**Table 1:** Baseline patient characteristics stratified by HER2 status (HER2 0 vs. HER2-low).

Demographics	Total (n = 1,433)	HER2 0 (n = 815)	HER2-low (n = 618)	*p* value*
Age (median)	49	49	49	0.23
<70 years	1,384 (96.6%)	783 (96.1%)	601 (97.2%)	
≥70 years	49 (3.4%)	32 (3.9%)	17 (2.8%)	
Performance status				0.32
0~1	1,356 (94.6%)	767 (94.1%)	589 (95.3%)	
≥2	77 (5.4%)	48 (5.9%)	29 (4.7%)	
Menopausal status^a^				0.96
Pre/peri-	833 (58.1%)	475 (58.3%)	358 (57.9%)	
Post-	579 (40.4%)	331 (40.6%)	248 (40.1%)	
Histology				**0.004**
Invasive ductal	1,297 (90.5%)	725 (89.0%)	572 (92.6%)	
Invasive lobular	76 (5.3%)	57 (7.0%)	19 (3.1%)	
Other	60 (4.2%)	32 (3.9%)	28 (4.5%)	
Nuclear grade^a^				0.07
I	20 (1.4%)	7 (0.9%)	13 (2.1%)	
II	324 (22.6%)	162 (19.9%)	162 (26.2%)	
III	185 (12.9%)	107 (13.1%)	78 (12.6%)	
Stage at diagnosis^a^				**<0.001**
I	125 (8.7%)	80 (9.8%)	45 (7.3%)	
II	466 (32.5%)	260 (31.9%)	206 (33.3%)	
III	351 (24.5%)	216 (26.5%)	135 (21.8%)	
IV	142 (9.9%)	55 (6.7%)	87 (14.1%)	
Ki-67^a^				0.24
Median (min–max)	30 (5–98)	30 (5–98)	30 (5–90)	
≤14%	149 (10.4%)	70 (8.6%)	79 (12.8%)	
>14%	465 (32.4%)	244 (29.9%)	221 (35.8%)	
Hormone receptor status				**<0.001**
Positive	1,045 (72.9%)	564 (69.2%)	481 (77.8%)	
Negative	388 (27.1%)	251 (30.8%)	137 (22.2%)	
Initial metastatic sites				0.22
Bone and soft tissue only	387 (27.0%)	232 (28.5%)	155 (25.1%)	
Liver	292 (20.4%)	157 (19.3%)	135 (21.8%)	
Lung	512 (35.7%)	302 (37.1%)	210 (34.0%)	
Number of metastatic sites^a^				0.53
<3	1,225 (85.5%)	701 (86.0%)	524 (84.8%)	
≥3	197 (13.7%)	108 (13.3%)	89 (14.4%)	
Disease-free interval in recurrent population (n = 1,291)				0.27
≤5 years	1,040 (72.6%)	620 (76.1%)	420 (68.0%)	
>5 years	251 (17.5%)	140 (17.2%)	111 (18.0%)	

a Some of the menopausal status, nuclear grade, clinical stage, Ki-67 index, and number of metastatic sites data were missing.

*Bold values indicate statistically significant results.

HER2, human epidermal growth factor receptor 2.

MBC patients with HER2-low tumors had a higher percentage of HR-positive disease compared to those with HER2-zero tumors (*p* < 0.001; **Figure 2**). In particular, patients with HER2 IHC2+ tumors showed the highest percentage (82.9%), followed by HER2 IHC1+ tumors (76.0%). When stratified by HR status, HER2-low tumors were more frequently presented in HR-positive group than that in HR-negative group. Specifically, the proportions of HER2 IHC1+ and IHC2+ tumors were 33.0% and 13.0% in HR-positive disease compared with 28.1% and 7.2% in HR-negative disease (*p* < 0.001; **Figure 2**). Other statistically significant differences between HER2-low tumors and HER2-zero tumors were detected for stage at primary breast cancer diagnosis (*p* < 0.001) and histology (*p* = 0.004). No differences were seen in proliferation rate (measured by Ki-67), menopausal status, and number of metastatic sites (**Table 1**).

When HER2-low tumors were further divided into HER2 IHC1+ and HER2 IHC2+ tumors, there were significant differences in HR status (*p* < 0.001), primary breast cancer diagnosis stage (*p* < 0.001), performance status (*p* = 0.002), histological grade (*p* = 0.015), and number of metastatic sites (*p* = 0.03) among HER2-zero, HER2 IHC1+, and HER2 IHC2+ tumor groups (**Supplementary Table S1**). When divided by HR status, HER2-low tumors had a higher percentage of stage IV disease compared to HER2-zero tumors in both HR-positive subgroup (*p* = 0.001) and HR-negative subgroup (*p* = 0.006). Fewer invasive lobular tumors were detected in HER2-low tumors compared to those in HER2-zero tumors in the HR-positive subgroup (*p* = 0.003). The baseline characteristics were otherwise similar between HER2-low and HER2-zero tumors based on HR status (**Supplementary Tables S2**, **S4**). When stratified by HER2 IHC status, a higher proportion of stage IV disease (*p* = 0.02) and fewer number of metastatic sites (*p* = 0.01) were detected for HER2 IHC2+ tumors compared to HER2 IHC0~1+ tumors in HR-positive population, while a higher Ki-67 index (*p* < 0.001) and more frequent disease relapse (*p* = 0.03) were observed for HER2 IHC2+ tumors in HR-negative population (**Supplementary Tables S3**, **S5**).

### Survival and Prognosis

**Figure 3:**
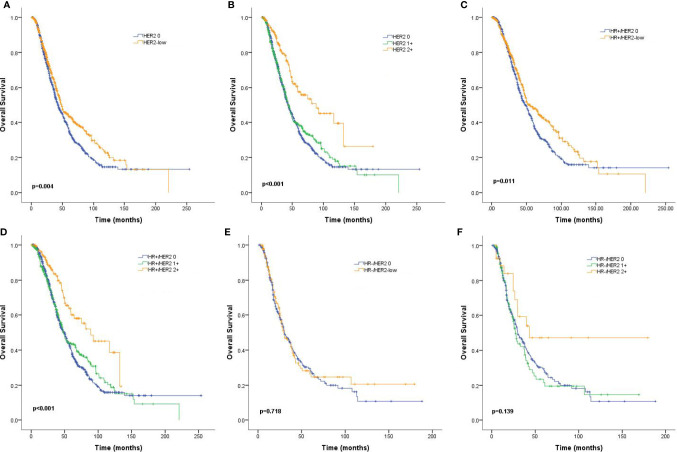
Kaplan–Meier curves for overall survival (OS) by hormone receptor (HR) status and human epidermal growth factor receptor 2 (HER2) immunohistochemistry (IHC) status. OS for HER2-low vs. HER2-zero tumors in the complete cohort **(A)**, HR-positive population **(C)**, and HR-negative population **(E)**, as well as OS curves for HER2 2+ vs. HER2 1+ vs. HER2-zero tumors for the complete cohort **(B)**, HR-positive population **(D)**, and HR-negative population **(F)**. *p* values are from the stratified log-rank test.

**Figure 4:**
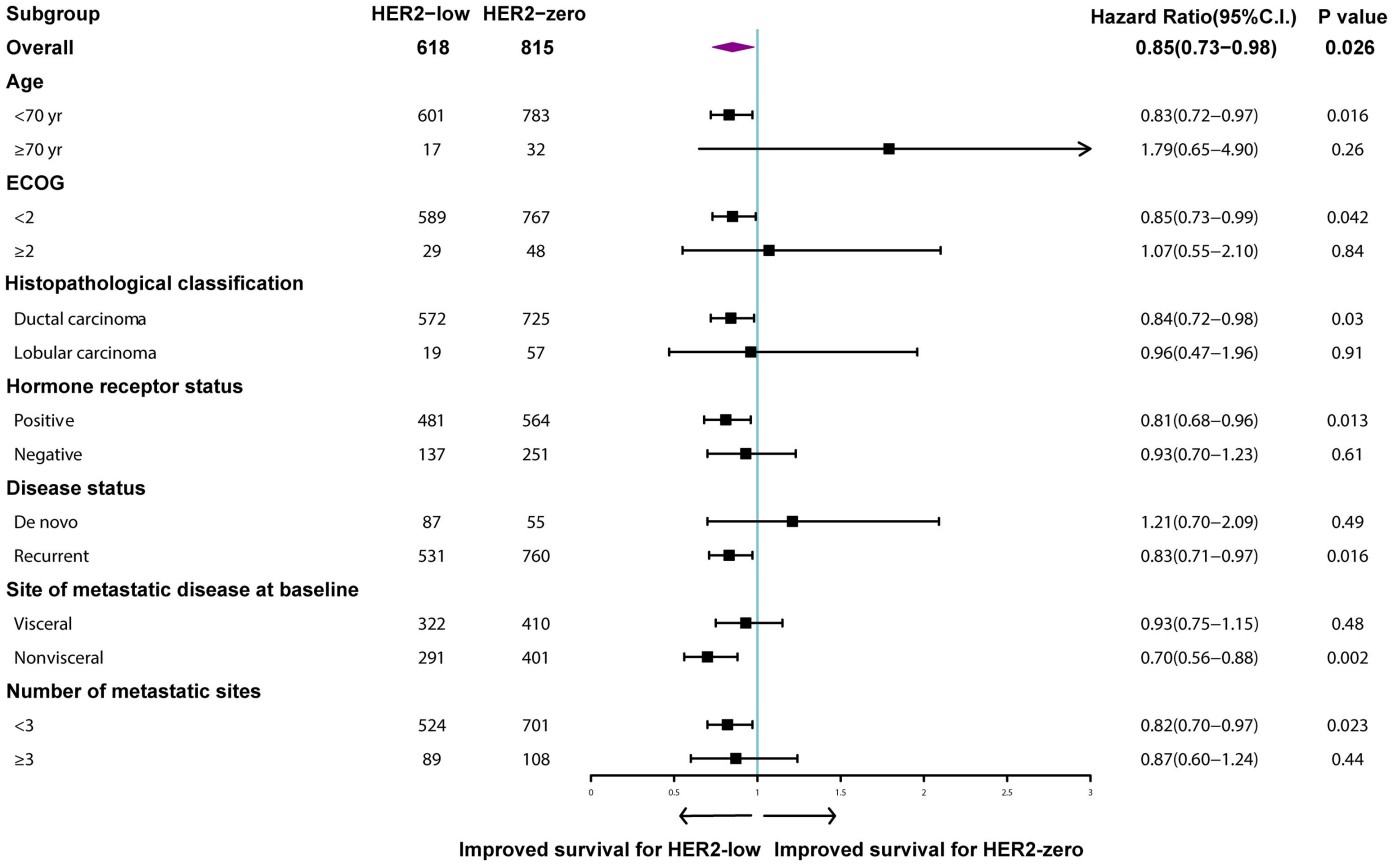
Multivariable Cox regression analysis for overall survival. Comparison of human epidermal growth factor receptor 2 (HER2)-low and HER2-zero breast cancer.

Median follow-up time for the entire population was 62.6 months (95% CI: 58.0–67.1). MBC patients with HER2-low tumors survived significantly longer than those with HER2-zero tumors (48.5 months vs. 43.0 months, *p* = 0.004; **Figure 3A**). A similar trend was observed for patients with HR-positive tumors (54.9 months vs. 48.1 months, *p* = 0.011; **Figure 3C**), but the difference was not statistically significant for the HR-negative subgroup (29.5 months vs. 29.9 months, *p* = 0.718; **Figure 3E**). More specifically, HER2 IHC2+ tumors had an improved OS compared to HER2 IHC1+ and HER2-zero tumors in the entire patient population (88.5 months vs. 43.6 months vs. 43.0 months, *p* < 0.001; **Figure 3B**) and the HR-positive subgroup (88.5 months vs. 47.7 months vs. 48.1 months, *p* < 0.001; **Figure 3D**), but not the HR-negative subgroup (42.9 months vs. 27.2 months vs. 29.9 months, *p* = 0.139; **Figure 3F**). Subgroup analyses revealed that the rates of OS were significantly different in patients with nonvisceral metastases (HR: 0.70, 95% CI: 0.56–0.88, *p* = 0.002) and positive HR status compared to their counterparts (HR: 0.81, 95% CI: 0.68–0.96, *p* = 0.013) (**Figure 4**).

**Table 2** summarizes the prognostic factors associated with OS in the HER2-negative population. HER2-low tumors were identified to be independently associated with increased OS (HR: 0.85, 95% CI: 0.73–0.98, *p* = 0.026), while HR-positive tumors (HR: 0.60, 95% CI: 0.51–0.70, *p* < 0.001), better performance status (HR: 0.75, 95% CI: 0.56–0.99, *p* = 0.039), and fewer number of metastatic sites (HR: 0.55, 95% CI: 0.46–0.67, *p* < 0.001) were significantly associated with improved OS (**Table 2**).

**Table 2:** Multivariate survival analysis following metastatic breast cancer diagnosis.

	Multivariate		
Factor	HR	95% CI	*p* value*
Age (<70/≥70)	0.80	0.56–1.14	0.22
ECOG (<2/≥2)	0.75	0.56–0.99	**0.039**
*De novo*/recurrent disease	1.00	0.76–1.30	0.97
Number of metastatic sites (<3/≥3)	0.55	0.46–0.67	**<0.001**
HER2-low/HER2 0	0.85	0.73–0.98	**0.026**
Hormone receptor positive/negative	0.60	0.51–0.70	**<0.001**

ECOG, Eastern Cooperative Oncology Group performance status; HR, hazard ratio; CI, confidence interval; HER2, human epidermal growth factor receptor 2.

*Bold values indicate statistically significant results.

## Discussion

To the best of our knowledge, this is the first and largest study that characterizes MBC patients with HER2-low tumors in China. The clinicopathological features and prognostic outcomes of HER2-negative MBC patients were analyzed to compare the differences between HER2-low and HER2-zero tumors. We found that HER2-low tumors accounted for almost half of HER2-negative tumors, and most of them occurred in patients with HR-positive disease. Moreover, HER2-low tumors differed distinctly from HER2-zero tumors in terms of HR positivity and survival. Notably, a particular survival benefit of HER2 IHC2+ tumors over HER2 IHC0~1+ tumors was observed in the entire population and HR-positive subgroup.

So far, only a few studies have investigated the proportions of HER2-low breast cancer within the HER2-negative population. In a retrospective study of 3,689 patients with HER2-negative disease, the percentages of HER2-low, HER2 IHC0, HER2 IHC1+, and HER2 IHC2+ patients were 59.7%, 40.4%, 40.3%, and 19.4%, respectively. However, a combination of multiple databases was used in this study, which extracted from studies with various inclusion/exclusion criteria; therefore, both early-stage and MBC patients were included and evaluation of HER2 IHC status was not standardized (15). A pooled analysis of 2,310 HER2-negative breast cancer patients from four prospective neoadjuvant clinical trials reported the proportion of HER2-low tumors as 47.5%, which was consistent with our results (16). However, this study included early-stage patients from different clinical trial cohorts, and heterogeneity among trials could not be avoided. Moreover, these two studies were limited to patients in western countries, and the results might not be that generalizable to Chinese populations. A retrospective study of 12,467 breast cancer patients from 19 Chinese clinical centers reported that the percentage of HER2-low tumors was 56.9%, but the rates varied among laboratories and some centers did not have standardized procedures for detecting HER2 status (17). Our results validated and added on to previous work by reporting the proportions of HER2-low, HER2 IHC0, HER2 IHC1+, and HER2 IHC2+ patients respectively among HER2-negative population based on the largest sample size in a single center study in China. As the National Cancer Center of China, our pathologists were well-qualified to perform HER2 testing. Moreover, in a single-center study, the heterogeneity of HER2 evaluation among laboratories was best avoided.

Another important finding of our study was that a significantly higher proportion of HR-positive disease was detected in patients with HER2-low tumors compared to those with HER2-zero tumors (77.8% vs. 69.2%, *p* < 0.001). The rate was remarkably high in the HER2 IHC2+ subgroup (82.9%). Moreover, a higher number of HER2-low tumors was found in HR-positive subgroup than those in HR-negative subgroup (46% vs. 35.3%, *p* < 0.001), suggesting an important role of HR expression in HER2-low tumors. Additionally, we observed that the majority (73.5%) of HER2-low patients had HER2 IHC1+ disease, regardless of HR status (33.0% in HR-positive subgroup vs. 28.1% in HR-negative subgroup, *p* = 0.75), which was in agreement with previous findings (15). The reasons for the differences in HR expression between HER2-low and HER2-zero tumors might be explained by the expression of individual genes. Schettini et al. (15) reported that luminal-related genes (e.g., BCL2, BAG1, FOXA1, ESR1) were significantly upregulated, while Basal-like genes and proliferation-related genes were significantly downregulated in HER2-low tumors compared to those in HER2-zero tumors. PAM50 analyses also showed that HER2-low tumors were characterized by Luminal A and B signatures, while Basal-like and normal-like subtypes were enriched in HER2-zero tumors. Gene expression profile identified HR status as the main driver of the underlying biology of HER2-low tumors. When stratified by HR status, the differences in subtype distribution and gene expression between HER2-low and HER2-zero tumors were consistently observed in HR-positive subgroup, while no obvious biological differences were detected in the HR-negative subgroup (15).

In addition to the pathological differences, a significantly improved OS of HER2-low tumors over HER2-zero tumors was found in the entire cohort. Cox regression analyses also identified HER2-low status as an independent prognostic factor associated with prolonged survival. Kaplan–Meier analyses revealed that the survival differences were especially relevant in the HR-positive subgroup but not the HR-negative subtype. Notably, when stratified by HER2 IHC status, the most significant survival benefit was observed in the HR+/HER2 IHC2+ subgroup (median survival: 88.5 months), while HR+/HER2 IHC1+ and HR+/HER2-zero patients had relatively similar median survival (43.6 months and 43.0 months, respectively).

The survival differences between HER2-low and HER2-zero tumors varied substantially across studies. Schettini et al. (15) found no differences between these two groups, and Denkert et al. (16) observed an improved 3-year disease-free survival and OS in early-stage HER2-low patients than that in HER2-zero patients. In this study, patients with HER2-low tumors were associated with better performance status, more frequent luminal disease, and a higher incidence of stage IV disease compared to those with HER2-zero tumors, suggesting that a reduced aggressiveness may explain their survival benefits. Besides, a reduced TP53 mutation rate and a higher expression of luminal-related genes had been reported in HER2-low and HR+/HER2-low subgroups, while a vast majority of proliferation-related genes and tyrosine-kinase receptor genes were detected in HER2-zero tumors, which might explain the improved survival of HER2-low tumors from a genomic background (15, 16). The fact that patients with HR+/HER2 IHC2+ tumors survived particularly longer than those with HR+/HER2 IHC0~1+ tumors in our study was somewhat intriguing. From the clinicopathological point of view, patients with HR+/HER2 2+tumors were younger, exhibited better performance status, and had fewer number of metastatic sites compared to those with HR+/HER2 0~1+ tumors, indicating a less advanced disease status and improved patient conditions that might explain their prolonged survival. Previous studies from western countries have reported a higher level of ERBB2 level in HR+/HER2 2+ tumors than that in HR+/HER2 1+ tumors, but the gene expression signatures between these two groups were otherwise similar (15). Next-generation was used as a robust tool to identify patients with HER2 amplification (n = 774) in China, and the results showed that HER2-low amplification patients had a distinct mutation profile from HER2 non-amplified patients (18). However, the authors did not further distinguish HER2 IHC2+ tumors from HER2 IHC1+tumors (18). Taken together, these findings suggested that HR is an essential determinant of the underlying biology of HER2-low tumors, and HR-positive/HER2-low subgroup might be identified as a more distinct biological entity within the HER2-negative population. Due to the retrospective nature of this study and the lack of genomic information, whether HR+/HER2 IHC2+ tumors could be further distinguished as a distinct subtype remains unknown. Further investigations aiming to characterize the more detailed molecular landscape of HER2-low population are warranted in the future. It is also essential to reveal the underlying molecular basis of the reduced prognosis of HER2-zero tumors and improved survival of HR+HER2-low/HER2 IHC2+ patients.

The results of our study not only add value to the general understanding of HER2-low disease but also have important implications for the development of new therapeutic strategies. The development of novel ADCs targeting HER2 has opened up a new window for the treatment of HER2-low breast cancer. Results from clinical trials of the most advanced ADC, T-DXd, have shown that even a low-to-moderate expression of HER2 receptor is sufficient to trigger therapy response (13). Interestingly, the treatment efficacy seemed to differ by HR status. In a phase Ib study of T-DXd, the objective response rate (ORR) of HR+ and HR- tumors was 40.4% and 14.3%, respectively, while the ORRs were similar between HER2 IHC1+ and HER2 IHC2+ groups (35.7% vs. 38.5%). Therefore, it might be inferred that HR+HER2-low disease is a more distinct subtype in the HER2-negative population. However, whether HER2 IHC status can affect therapeutic responses and survival benefits remains undetermined, and the results of the ongoing phase III trial DESTINY-Breast 04 (NCT03734029) focusing specifically on HER2-low MBC patients might give us an answer in the near future.

Our findings confirmed the fact that the subgroups of HER2-low tumors could be distinguished according to the current ASCO/CAP guidelines for evaluation of HER2 expression. Nevertheless, substantial heterogeneity still exists, and further standardization is needed to help understand the detailed molecular landscape of HER2-low breast tumors. Besides, the inconsistency of HER2 IHC detection between different laboratories should be addressed. Previous studies have reported the discordance rates of 30% for HER2 IHC1+ and 60% for HER2 IHC2+. For HER2 IHC1+ vs. HER2 IHC0, almost 50% discordance have been identified (13, 15). Furthermore, as HER2 IHC1+ had no significant clinical implications previously, it was frequently combined with HER2 IHC0. Therefore, there is an urgent need to train pathologists and to develop more sensitive assays for HER2 evaluation, including mRNA expression and next-generation sequencing.

Our study has several limitations. First, this was a retrospective single-center study; thus, some imbalances between groups and referral bias might exist. However, the intra-laboratory heterogeneity of HER2 detection was somewhat avoided. Second, the HER2 IHC status was evaluated based on the primary tumor. Rebiopsy of metastatic lesions was not performed in the majority of patients, and the discordance of HER2 status could not be ruled out. Third, our study did not include biological information of HER2-low patients. Large-scale genomic analyses might shed some light on the genomic background of HER2-low patients in the near future.

## Conclusion

Our study provides new insight into the clinicopathological features, survival, and prognosis of HER2-low tumors within the HER2-negative population in MBC patients in China. We found that HER2-low tumors could be identified as a more distinct clinical entity from HER2-zero tumors, especially for the HR-positive subgroup. A more complex molecular landscape of HER2-low breast cancer might exist, and novel sensitive assays that can further distinguish HER2-levels could be investigated, thus offering new therapeutic targets for breast cancer treatment.

## Data Availability Statement

The original contributions presented in the study are included in the article/**Supplementary Material**. Further inquiries can be directed to the corresponding author.

## Ethics Statement

The studies involving human participants were reviewed and approved by the National Cancer Center/National Clinical Research Center for Cancer/Cancer Hospital, Chinese Academy of Medical Sciences and Peking Union Medical College. Written informed consent for participation was not required for this study in accordance with the national legislation and the institutional requirements.

## Author Contributions

YQL and NA: article writing, data collection and analysis. HM, XG, SL, ZW, YC: data extraction. SC, QiaL, RC, JW, YL, YF, PY, PZ, QinL, FM: management of patients and data collection. BX: study design and supervision. All authors have read and approved this article and have agreed to its submittal to this journal.

## Conflict of Interest

The authors declare that the research was conducted in the absence of any commercial or financial relationships that could be construed as a potential conflict of interest.

## Publisher’s Note

All claims expressed in this article are solely those of the authors and do not necessarily represent those of their affiliated organizations, or those of the publisher, the editors and the reviewers. Any product that may be evaluated in this article, or claim that may be made by its manufacturer, is not guaranteed or endorsed by the publisher.
