# Supplementary material for: In Real Life, Low-Level HER2 Expression May Be Associated With Better Outcome in HER2-Negative Breast Cancer: A Study of the National Cancer Center, China
Source: Front Oncol. 2022 Jan 17;11:774577. doi: 10.3389/fonc.2021.774577 (PMC8801428; doi:10.3389/fonc.2021.774577)
Supplement: Supplementary file 1 [file Table_1.docx]

Table 1. Baseline patient characteristics stratified by HER2 status

(HER2 0 vs. HER2 1+ vs. HER2 2+)

| Demographics | Total  (n=1433) | HER2 0  (n=815) | HER2 1+  (n=454) | HER2 2+  (n=164) | p value* |
| --- | --- | --- | --- | --- | --- |
| Age (median) | 49 | 49 | 49 | 49 |  |
| <70 years | 1384 (96.6%) | 783 (96.1%) | 439 (96.7%) | 162 (98.8%) | 0.22 |
| ≥70 years | 49 (3.4%) | 32 (3.9%) | 15 (3.3%) | 2 (1.2%) |  |
| Performance Status |  |  |  |  | **0.002** |
| 0~1 | 1356 (94.6%) | 767 (94.1%) | 429 (94.5%) | 160 (97.6%) |  |
| ≥2 | 77 (5.4%) | 33 (4.0%) | 39 (8.6%) | 5 (3.0%) |  |
| Menopausal Status^a^ |  |  |  |  | 0.68 |
| Pre/peri- | 833 (58.1%) | 475 (58.3%) | 267 (58.8%) | 91 (55.5%) |  |
| Post- | 579 (40.4%) | 331 (40.6%) | 177 (39.0%) | 71 (43.3%) |  |
| Histology |  |  |  |  | **0.015** |
| Invasive ductal | 1297 (90.5%) | 725 (89.0%) | 422 (93.0%) | 150 (91.5%) |  |
| Invasive lobular | 76 (5.3%) | 57 (7.0%) | 14 (3.1%) | 5 (3.0%) |  |
| Other | 60 (4.2%) | 32 (3.9%) | 18 (4.0%) | 10 (6.1%) |  |
| Nuclear Grade^a^ |  |  |  |  | 0.15 |
| I | 20 (1.4%) | 7 (0.9%) | 8 (1.8%) | 5 (3.0%) |  |
| II | 324 (22.6%) | 162 (19.9%) | 119 (26.2%) | 43 (26.2%) |  |
| III | 185 (12.9%) | 107 (13.1%) | 54 (11.9%) | 24 (14.6%) |  |
| Stage at diagnosis^a^ |  |  |  |  | **<0.001** |
| I | 125 (8.7%) | 80 (9.8%) | 29 (6.4%) | 16 (9.8%) |  |
| II | 466 (32.5%) | 260 (31.9%) | 155 (34.1%) | 51 (31.1%) |  |
| III | 351 (24.5%) | 216 (26.5%) | 100 (22.0%) | 35 (21.3%) |  |
| IV | 142 (9.9%) | 55 (6.7%) | 58 (12.8%) | 29 (17.7%) |  |
| Ki-67^a^ |  |  |  |  | 0.20 |
| Median (min-max) | 30 (5-98) | 30 (5-98) | 30 (5-90) | 30 (10-90) |  |
| ≤14% | 149 (10.4%) | 70 (8.6%) | 46 (10.1%) | 33 (20.1%) |  |
| >14% | 465 (32.4%) | 244 (29.9%) | 147 (32.4%) | 74 (45.1%) |  |
| Hormone receptor status |  |  |  |  | **<0.001** |
| Positive | 1045 (72.9%) | 564 (69.2%) | 345 (76.0%) | 136 (82.9%) |  |
| Negative | 388 (27.1%) | 251 (30.8%) | 109 (24.0%) | 28 (17.1%) |  |
| Initial metastatic sites |  |  |  |  | 0.34 |
| Bone and soft tissue only | 387 (27.0%) | 232 (28.5%) | 111 (24.4%) | 44 (26.8%) |  |
| Liver | 292 (20.4%) | 157 (19.3%) | 98 (21.6%) | 37 (22.6%) |  |
| Lung | 512 (35.7%) | 302 (37.1%) | 163 (35.9%) | 47 (28.7%) |  |
| Number of metastatic sites^a^ |  |  |  |  | **0.03** |
| < 3 | 1225 (85.5%) | 701 (86.0%) | 375 (82.6%) | 149 (90.9%) |  |
| ≥ 3 | 197 (13.7%) | 108 (13.3%) | 75 (16.5%) | 14 (8.5%) |  |
| Disease-free interval in recurrent population (n=1291) |  |  |  |  | 0.23 |
| ≤ 5 years | 1040 (72.6%) | 620 (76.1%) | 308 (67.8%) | 112 (68.3%) |  |
| > 5 years | 251 (17.5%) | 140 (17.2%) | 88 (19.4%) | 23 (14.0%) |  |

^a^Some of menopausal status, nuclear grades, clinical stage, Ki-67 index and number of metastatic sites information were missing.

*Χ^2^ or Fisher’s exact test. Bold values indicate statistically significant results.
