# Supplementary material for: In Real Life, Low-Level HER2 Expression May Be Associated With Better Outcome in HER2-Negative Breast Cancer: A Study of the National Cancer Center, China
Source: Front Oncol. 2022 Jan 17;11:774577. doi: 10.3389/fonc.2021.774577 (PMC8801428; doi:10.3389/fonc.2021.774577)
Supplement: Supplementary file 2 [file Table_2.docx]

Table 2. Baseline patient characteristics stratified by HER2 status in HR-positive subgroup

(HER2 0 vs. HER2-low)

| Demographics | Total | HER2 0 | HER2-low | p value* |
| --- | --- | --- | --- | --- |
|  | (n=1045) | (n=564) | (n=481) |  |
| Age (median) | 49 | 49 | 49 |  |
| <70 years | 1009 (96.6%) | 543 (96.3%) | 466 (96.9%) | 0.59 |
| ≥70 years | 36 (3.4%) | 21 (3.7%) | 15 (3.1%) |  |
| Performance Status |  |  |  | 0.91 |
| 0~1 | 992 (94.9%) | 535 (94.9%) | 457 (95.0%) |  |
| ≥2 | 53 (5.1%) | 29 (5.1%) | 24 (5.0%) |  |
| Menopausal Status^a^ |  |  |  | 0.57 |
| Pre/peri- | 596 (57.0%) | 319 (56.6%) | 277 (57.6%) |  |
| Post- | 432 (41.3%) | 239 (42.4%) | 193 (40.1%) |  |
| Histology |  |  |  | **0.003** |
| Invasive ductal | 933 (89.3%) | 496 (87.9%) | 437 (90.9%) |  |
| Invasive lobular | 66 (6.3%) | 48 (8.5%) | 18 (3.7%) |  |
| Other | 46 (4.4%) | 20 (3.5%) | 26 (5.4%) |  |
| Nuclear Grade^a^ |  |  |  | 0.43 |
| I | 18 (1.7%) | 7 (1.2%) | 11 (2.3%) |  |
| II | 257 (24.6%) | 121 (21.5%) | 136 (28.3%) |  |
| III | 104 (10.0%) | 55 (9.8%) | 49 (10.2%) |  |
| Stage at diagnosis^a^ |  |  |  | **0.001** |
| I | 91 (8.7%) | 52 (9.2%) | 39 (8.1%) |  |
| II | 351 (33.6%) | 194 (34.4%) | 157 (32.6%) |  |
| III | 236 (22.6%) | 135 (23.9%) | 101 (21.0%) |  |
| IV | 111 (10.6%) | 40 (7.1%) | 71 (14.8%) |  |
| Ki-67^a^ |  |  |  | 0.81 |
| Median (min-max) | 30 (5-98) | 30 (5-98) | 25 (5-80) |  |
| ≤14% | 123 (11.8%) | 57 (10.1%) | 66 (13.7%) |  |
| >14% | 315 (30.1%) | 150 (26.6%) | 165 (34.3%) |  |
| Initial metastatic sites |  |  |  | 0.46 |
| Bone and soft tissue only | 299 (28.6%) | 171 (30.3%) | 128 (26.6%) |  |
| Liver | 229 (21.9%) | 120 (21.3%) | 109 (22.7%) |  |
| Lung | 343 (32.8%) | 196 (34.8%) | 147 (30.6%) |  |
| Number of metastatic sites^a^ |  |  |  | 0.63 |
| < 3 | 880 (84.2%) | 472 (83.7%) | 408 (84.8%) |  |
| ≥ 3 | 158 (15.1%) | 88 (15.6%) | 70 (14.6%) |  |
| Disease-free interval in recurrent population (n=935) |  |  |  | 0.55 |
| ≤ 5 years | 718 (68.7%) | 407 (72.2%) | 311 (64.7%) |  |
| > 5 years | 217 (20.8%) | 118 (20.9%) | 99 (20.6%) |  |

^a^Some of menopausal status, nuclear grades, clinical stage, Ki-67 index and number of metastatic sites information were missing.

HR: hormone receptor

*Χ^2^ or Fisher’s exact test. Bold values indicate statistically significant results.
