# Supplementary material for: In Real Life, Low-Level HER2 Expression May Be Associated With Better Outcome in HER2-Negative Breast Cancer: A Study of the National Cancer Center, China
Source: Front Oncol. 2022 Jan 17;11:774577. doi: 10.3389/fonc.2021.774577 (PMC8801428; doi:10.3389/fonc.2021.774577)
Supplement: Supplementary file 3 [file Table_3.docx]

Table 3. Baseline patient characteristics stratified by HER2 status in HR-positive subgroup

(HER2 0 and 1+ vs. HER2 2+)

| Demographics | Total | HER2 0 and 1+ | HER2 2+ | p value* |
| --- | --- | --- | --- | --- |
|  | (n=1045) | (n=909) | (n=136) |  |
| Age (median) | 49 | 49 | 49 |  |
| <70 years | 1009 (96.6%) | 874 (96.1%) | 135 (99.3%) | 0.08 |
| ≥70 years | 36 (3.4%) | 35 (3.9%) | 1 (0.7%) |  |
| Performance Status |  |  |  | 0.30 |
| 0~1 | 992 (94.9%) | 860 (94.6%) | 132 (97.1%) |  |
| ≥2 | 53 (3.3%) | 49 (5.4%) | 4(2.9%) |  |
| Menopausal Status^a^ |  |  |  | 0.54 |
| Pre/peri- | 596 (57.0%) | 521 (57.3%) | 75 (55.1%) |  |
| Post- | 432 (41.3%) | 372 (40.9%) | 60 (44.1%) |  |
| Histology |  |  |  | 0.28 |
| Invasive ductal | 933 (89.3%) | 809 (89.0%) | 124 (91.2%) |  |
| Invasive lobular | 66 (6.3%) | 61 (6.7%) | 5 (3.7%) |  |
| Other | 46 (4.4%) | 38 (4.2%) | 8 (5.9%) |  |
| Nuclear Grade^a^ |  |  |  | 0.67 |
| I | 18 (1.7%) | 14 (1.5%) | 4 (2.9%) |  |
| II | 257 (24.6%) | 217 (23.9%) | 40 (29.4%) |  |
| III | 104 (10.0%) | 89 (9.8%) | 15 (11.0%) |  |
| Stage at diagnosis^a^ |  |  |  | **0.02** |
| I | 91 (8.7%) | 76 (8.4%) | 15 (11.0%) |  |
| II | 351 (33.6%) | 310 (34.1%) | 41 (30.1%) |  |
| III | 236 (22.6%) | 209 (23.0%) | 27 (19.9%) |  |
| IV | 111 (10.6%) | 86 (9.5%) | 25 (18.4%) |  |
| Ki-67^a^ |  |  |  | 0.68 |
| Median (min-max) | 30 (5-98) | 25 (5-98) | 25 (5-70) |  |
| ≤14% | 123 (11.8%) | 97 (10.7%) | 26 (19.1%) |  |
| >14% | 315 (30.1%) | 254 (27.9%) | 61 (44.9%) |  |
| Initial metastatic sites |  |  |  | 0.38 |
| Bone and soft tissue only | 299 (28.6%) | 262 (28.8%) | 37 (27.2%) |  |
| Liver | 229 (21.9%) | 197 (21.7%) | 32 (23.5%) |  |
| Lung | 343 (32.8%) | 308 (33.9%) | 35 (25.7%) |  |
| Number of metastatic sites^a^ |  |  |  | **0.01** |
| < 3 | 880 (84.2%) | 756 (83.2%) | 124 (91.2%) |  |
| ≥ 3 | 158 (15.1%) | 147 (16.2%) | 11 (8.1%) |  |
| Disease-free interval in recurrent population (n=935) |  |  |  | 0.25 |
| ≤ 5 years | 718 (68.7%) | 628 (69.1%) | 90 (66.2%) |  |
| > 5 years | 217 (20.8%) | 196 (21.6%) | 21 (15.4%) |  |

^a^Some of menopausal status, nuclear grades, clinical stage, Ki-67 index and number of metastatic sites information were missing.

HR: hormone receptor;

*Χ^2^ or Fisher’s exact test. Bold values indicate statistically significant results.
