# Supplementary material for: In Real Life, Low-Level HER2 Expression May Be Associated With Better Outcome in HER2-Negative Breast Cancer: A Study of the National Cancer Center, China
Source: Front Oncol. 2022 Jan 17;11:774577. doi: 10.3389/fonc.2021.774577 (PMC8801428; doi:10.3389/fonc.2021.774577)
Supplement: Supplementary file 4 [file Table_4.docx]

Table 4. Baseline patient characteristics stratified by HER2 status in HR-negative subgroup

(HER2 0 vs. HER2-low)

| Demographics | Total | HER2 0 | HER2-low | p value* |
| --- | --- | --- | --- | --- |
|  | (n=388) | (n=251) | (n=137) |  |
| Age (median) | 49 | 49 | 49 |  |
| <70 years | 375 (96.6%) | 240 (95.6%) | 135 (98.5%) | 0.15 |
| ≥70 years | 13 (3.4%) | 11 (4.4%) | 2 (1.5%) |  |
| Performance Status |  |  |  | 0.13 |
| 0~1 | 364 (93.8%) | 232 (92.4%) | 132 (96.4%) |  |
| ≥2 | 24 (6.2%) | 19 (7.6%) | 5 (3.6%) |  |
| Menopausal Status^a^ |  |  |  | 0.52 |
| Pre/peri- | 237 (61.1%) | 156 (62.2%) | 81 (59.1%) |  |
| Post- | 147 (37.9%) | 92 (36.7%) | 55 (40.1%) |  |
| Histology |  |  |  | 0.051 |
| Invasive ductal | 364 (93.8%) | 229 (91.2%) | 135 (98.5%) |  |
| Invasive lobular | 10 (2.6%) | 9 (3.6%) | 1 (0.7%) |  |
| Other | 14 (3.6%) | 12 (4.8%) | 2 (1.5%) |  |
| Nuclear Grade^a^ |  |  |  | 0.20 |
| I | 2 (0.5%) | 0 (0.0%) | 2 (1.5%) |  |
| II | 67 (17.3%) | 41 (16.3%) | 26 (19.0%) |  |
| III | 81 (20.9%) | 52 (20.7%) | 29 (21.2%) |  |
| Stage at diagnosis^a^ |  |  |  | **0.006** |
| I | 34 (8.8%) | 28 (11.2%) | 6 (4.4%) |  |
| II | 115 (29.6%) | 66 (26.3%) | 49 (35.8%) |  |
| III | 115 (29.6%) | 81 (32.3%) | 34 (24.8%) |  |
| IV | 31 (8.0%) | 15 (6.0%) | 16 (11.7%) |  |
| Ki-67^a^ |  |  |  | 0.22 |
| Median (min-max) | 50 (5-90) | 60 (5-90) | 40 (5-90) |  |
| ≤14% | 26 (6.7%) | 13 (5.2%) | 13 (9.5%) |  |
| >14% | 150 (38.7%) | 94 (37.5%) | 56 (40.9%) |  |
| Initial metastatic sites |  |  |  | 0.38 |
| Bone and soft tissue only | 88 (22.7%) | 61 (24.3%) | 27 (19.7%) |  |
| Liver | 63 (16.2%) | 37 (14.7%) | 26 (19.0%) |  |
| Lung | 169 (43.6%) | 106 (42.2%) | 63 (46.0%) |  |
| Number of metastatic sites^a^ |  |  |  | 0.06 |
| < 3 | 345 (88.9%) | 229 (91.2%) | 116 (84.7%) |  |
| ≥ 3 | 39 (10.1%) | 20 (8.0%) | 19 (13.9%) |  |
| Disease-free interval in recurrent population (n=356) |  |  |  | 0.87 |
| ≤ 5 years | 322 (83.0%) | 213 (84.9%) | 109 (79.6%) |  |
| > 5 years | 34 (8.8%) | 22 (8.8%) | 12 (8.8%) |  |

^a^Some of menopausal status, nuclear grades, clinical stage, Ki-67 index and number of metastatic sites information were missing.

HR: hormone receptor.

*Χ^2^ or Fisher’s exact test. Bold values indicate statistically significant results.
