# Supplementary material for: In Real Life, Low-Level HER2 Expression May Be Associated With Better Outcome in HER2-Negative Breast Cancer: A Study of the National Cancer Center, China
Source: Front Oncol. 2022 Jan 17;11:774577. doi: 10.3389/fonc.2021.774577 (PMC8801428; doi:10.3389/fonc.2021.774577)
Supplement: Supplementary file 5 [file Table_5.docx]

Table 5. Baseline patient characteristics stratified by HER2 status in HR-negative subgroup

(HER2 0 and 1+ vs. HER2 2+)

| Demographics | Total | HER2 0 and 1+ | HER2 2+ | p value* |
| --- | --- | --- | --- | --- |
|  | (n=388) | (n=360) | (n=28) |  |
| Age (median) | 49 | 49 | 49 |  |
| <70 years | 375 (96.6%) | 348 (96.7%) | 27 (96.4%) | 1.00 |
| ≥70 years | 13 (3.4%) | 12 (3.3%) | 1 (3.6%) |  |
| Performance Status |  |  |  | 0.38 |
| 0~1 | 364 (93.8%) | 336 (93.3%) | 28 (100.0%) |  |
| ≥2 | 24 (6.2%) | 24 (6.7%) | 0 (0.0%) |  |
| Menopausal Status^a^ |  |  |  | 0.79 |
| Pre/peri- | 237 (61.1%) | 221 (61.4%) | 16 (57.1%) |  |
| Post- | 147 (37.9%) | 136 (37.8%) | 11 (39.3%) |  |
| Histology |  |  |  |  |
| Invasive ductal | 364 (93.8%) | 338 (93.9%) | 26 (92.9 %) | 0.39 |
| Invasive lobular | 10 (2.6%) | 10 (2.8%) | 0 (0.0%) |  |
| Other | 14 (3.6%) | 12 (3.3%) | 2 (7.1%) |  |
| Nuclear Grade^a^ |  |  |  |  |
| I | 2 (0.5%) | 1 (0.3%) | 1 (3.6%) | 0.06 |
| II | 67 (17.3%) | 64 (17.8%) | 3 (10.7%) |  |
| III | 81 (20.9%) | 72 (20.0%) | 9 (32.1%) |  |
| Stage at diagnosis^a^ |  |  |  | 0.50 |
| I | 34 (8.8%) | 33 (9.2%) | 1 (3.6%) |  |
| II | 115 (29.6%) | 105 (29.2%) | 10 (35.7%) |  |
| III | 115 (29.6%) | 107 (29.7%) | 8 (28.6%) |  |
| IV | 31 (8.0%) | 27 (7.5%) | 4 (14.3%) |  |
| Ki-67^a^ |  |  |  | **<0.001** |
| Median (min-max) | 50 (5-90) | 50 (5-90) | 40 (5-90) |  |
| ≤14% | 26 (6.7%) | 19 (5.3%) | 7 (25.0%) |  |
| >14% | 150 (38.7%) | 137 (38.1%) | 13 (46.4%) |  |
| Initial metastatic sites |  |  |  | 0.96 |
| Bone and soft tissue only | 88 (22.7%) | 81 (22.5%) | 7 (25.0%) |  |
| Liver | 63 (16.2%) | 58 (16.1%) | 5 (17.9%) |  |
| Lung | 169 (43.6%) | 157 (43.6%) | 12 (42.9%) |  |
| Number of metastatic sites^a^ |  |  |  | 1.00 |
| < 3 | 345 (88.9%) | 320 (88.9%) | 25 (89.3%) |  |
| ≥ 3 | 39 (10.1%) | 36 (10.0%) | 3 (10.7%) |  |
| Disease-free interval in recurrent population (n=356) |  |  |  | **0.03** |
| ≤ 5 years | 322 (83.0%) | 300 (83.3%) | 22 (78.6%) |  |
| > 5 years | 34 (8.8%) | 32 (8.9%) | 2 (7.1%) |  |

^a^Some of menopausal status, nuclear grades, clinical stage, Ki-67 index and number of metastatic sites information were missing.

HR: hormone receptor

*Χ^2^ or Fisher’s exact test. Bold values indicate statistically significant results.
